# Exploring Patient Understandings of Navigation Services Within Alberta's Healthcare System: A Qualitative Study

**DOI:** 10.1111/hex.70383

**Published:** 2025-08-11

**Authors:** Sarah Rabi, Maria Santana, Gina Dimitropoulos, Kerry McBrien, Eleanor Benterud, Lorraine Wigston, Karen Tang

**Affiliations:** ^1^ Department of Community Health Sciences University of Calgary Calgary Alberta Canada; ^2^ O'Brien Institute for Public Health University of Calgary Calgary Alberta Canada; ^3^ Patient Engagement Team, Alberta Strategy for Patient‐Oriented Research Calgary Alberta Canada; ^4^ Department of Pediatrics University of Calgary Calgary Alberta Canada; ^5^ Mathison Centre for Mental Health Research & Education, Foothills Medical Centre Calgary Alberta Canada; ^6^ Hotchkiss Brain Institute University of Calgary Calgary Alberta Canada; ^7^ Faculty of Social Work University of Calgary Calgary Alberta Canada; ^8^ Department of Family Medicine University of Calgary Calgary Alberta Canada; ^9^ Department of Medicine University of Calgary Calgary Alberta Canada

**Keywords:** health services research, interpretive description, patient experience, patient navigation, patient‐oriented research

## Abstract

**Introduction:**

Patient navigation was first envisioned to assist marginalized cancer patients access timely healthcare services by identifying and addressing social barriers to care. While this understanding of patient navigation may still hold for a subgroup of programs today, its expansion over the past 30 years has resulted in a diverse set of interventions with distinct care settings, patient eligibility criteria, navigator training requirements and program goals. This study aimed to explore patients' understanding of patient navigation programs to identify program features that are of particular value and importance to them.

**Methods:**

In this qualitative study, we conducted one‐on‐one semi‐structured interviews from November 2023 to February 2024 with patients involved in five distinct hospital‐, clinic‐ and community‐based patient navigation programs across Alberta. Inductive thematic analysis and interpretive exercises were performed to construct a coherent narrative relevant to the research objective. Study participants were adult patients with patient navigation program exposure for at least 1 month (range: 2 months to 11 years).

**Results:**

Twenty‐three patient experiences were captured in the study (12 [52%] women; median [IQR] age, 59 [48–67] years), with approximately half receiving support from a nurse navigator (11/23, 48%). Regardless of navigation type, the patients' stories were tethered by their navigators' provision of personalized, seamless and humanized care. These perceived navigator functions were accomplished through patient‐identified navigator characteristics, including navigator approachability, accessibility and comprehensive systems knowledge. While the identified functions and characteristics of navigators were consistent across patients, the operationalization of these components varied based on the program's setting and the particular needs of each patient.

**Conclusions:**

The commonalities in patient perceptions of patient navigation indicate continued points of overlap across programs despite their increasing heterogeneity. Additionally, our findings provide insight into the functions and characteristics of patient navigation most valued by patients, which may inform future program development and implementation efforts.

**Patient and Public Contribution:**

Continued collaboration with two patient partners was maintained throughout the study to ensure responsiveness to patient priorities.

## 1 Introduction

Patient navigation (PN) was first envisioned in 1990 as a complex health intervention to support patients facing disproportionate barriers to timely breast cancer treatment in Harlem, New York [1]. In the inaugural PN program, lay (i.e., non‐clinically trained) navigators were tasked with supporting patients following the receipt of a suspicious breast cancer screening finding by working to address financial, informational, logistical, emotional or other barriers to care until the point of diagnostic resolution was achieved [2, 3, 4]. Following program implementation, early breast cancer detection at stages 0‐1 in the Harlem Hospital increased from 6% to 41% and 5‐year survival rose from 39% to 70% [5].

Recognizing the implications of PN, international interest in the intervention has led to widespread uptake and clinical expansion of PN programming beyond the confines of cancer treatment [6], with programs emerging in areas of chronic disease management [7, 8, 9], mental health [10], emergency care [11, 12], primary care [13] and paediatric‐to‐adult care transitions [14, 15, 16]. This broadening in PN scope has also led to the appointment of navigators with different training backgrounds (e.g., nurses, social workers, pharmacists and peer navigators) [17, 18]. While the adaptability of PN programs to different patient contexts and needs is critical, as is the case with any complex health intervention, there ought to remain some irreducible elements of PN that are visible across all programs [19, 20]. It appears, however, that as literature on PN proliferates, these elements have become increasingly difficult to pinpoint [21]. For example, it seems unlikely that a program consisting of lay navigators who link patients struggling with poor mental health to community‐based health and social services would have much conceptual overlap with another program composed of oral oncology nurse navigators who work to support symptom management and patient adherence during chemotherapy treatments [10, 22]. Such a lack of consensus surrounding PN conceptualization poses issues for effective program development and capacity building (e.g., navigator training) [23]. Furthermore, without a universal understanding of PN and navigator roles, researchers and policymakers face significant challenges when attempting to evaluate, replicate and/or scale such interventions [24].

It remains unclear how such program heterogeneity has impacted patients' experiences with and understandings of PN. Prior studies examining patients' experiences with program‐ or condition‐specific PN services have proposed overarching navigator functions related to the provision of comprehensive care [16, 25, 26, 27, 28, 29], explaining pertinent clinical information [12, 16, 25, 26, 28, 30, 31, 32], improving patient self‐efficacy [12, 16, 25, 26, 29, 31, 33], mitigating health‐related social needs (e.g., food access, transportation, housing, caregiver responsibilities) [12, 16, 25, 29, 33], bridging structural gaps in patient care [12, 16, 25, 27, 28, 29, 30, 31, 32, 33], scheduling medical appointments [12, 16, 25, 31, 33] and cultivating strong client‐navigator relationships [12, 16, 25, 26, 27, 28, 29, 30, 31, 32, 33]. While this study provides insightful context‐specific information, the integration of patient perspectives across distinct PN programs is needed to develop a more global understanding of PN [34, 35].

This qualitative study, therefore, sought to explore patients' understanding of distinct PN programs to identify shared PN functions and characteristics felt to be of particular value and importance to patients. Recognizing that PN programs constitute complex interventions where context‐level adaptations are critical to their success, the focus of this study was not per se on *what* individual programs did but on *how* they affected care recipients [36]. As such, utilizing patient experiences with PN served as an essential medium through which to address the study's aim.

## 2 Materials and Methods

Interpretive description (ID) was used as the methodological approach for this study [37]. ID is a qualitative research methodology designed to generate practical, contextually grounded knowledge relevant to applied health disciplines [38]. Its flexible and non‐prescriptive nature allows researchers to critically engage with disciplinary logic, selecting approaches that align best with their research question and support meaningful applications in real‐world settings [38, 39]. To do so, ID necessitates inductive approaches to data analysis that are concurrent with data collection, ensuring that all study findings are developed within the context of the data itself [38].

Employing ID, this study sought to distill a common understanding of the essential functions and characteristics of PN, while not assuming that the findings generated would be comprehensive of all patient experiences with PN. Throughout the study, particular interest was given to ensuring that the research process was guided by a clear and consistent approach to knowledge, that patients' experiences with PN were accurately represented, that data analysis was logical and transparent, and that our interpretations were well‐supported and trustworthy [38, 40]. The promotion of representative credibility and analytic logic in this project was achieved through maximum variation sampling (see Study Participants section below) and the construction of an audit trail, respectively. Interpretive authority was also bolstered through the use of thick description (that which goes beyond superficial accounts of participant responses to explore their underlying context or meaning) and the input of patient partners, who provided a mechanism to check for interpretation accuracy [41].

### 2.1 Patient Engagement Strategy

Recognizing the importance and need for patient‐oriented health research, the research team incorporated the perspectives of two dedicated patient partners (EB, LW) who served as co‐investigators in the study. Monthly meetings were held with the patient partners throughout the study to ensure our methodologies and questions aligned with the interests of patients in the province. In these meetings, the patient partners helped codevelop the interview guide and drove priority setting [42]. They also provided critical feedback on a range of other research activities, including the generation of the codebook and final themes, manuscript revisions and knowledge translation efforts.

### 2.2 Study Participants

Patients (or ‘clients’, as recipients of PN programs may alternatively be referred) were recruited from active Albertan PN programs. Maximum variation purposive sampling was performed to recruit a diverse participant sample across three strata (i.e., gender, socioeconomic status and navigation type), selected for their foreseeable implications on PN programming and service reliance [43, 44, 45]. In an effort to learn from patients with differing PN experiences, no restrictions were placed on the clinical scope of navigation services offered by PN programs or the training of their navigators. PN programs were excluded from the study if they were not patient‐facing (i.e., only offered referral‐based services) or if they were privately operated. Additionally, we did not include PN programs that specifically served Indigenous communities, recognizing the distinct roles these programs serve in addressing the historical and ongoing impacts of colonialism in Western healthcare systems [46]. The cultural significance of Indigenous PN programs could not be adequately explored within the scope of this current study, especially without Indigenous voices on our study team [47]. Instead, a separate study, partnered with Indigenous community members, is needed to highlight the unique cultural considerations of these programs.

Tang et al.'s [48] environmental scan of PN programs in the province provided a working list of potential recruitment sources that were further verified and expanded through a secondary search using publicly available program websites, an Alberta Health Services directory (https://www.albertahealthservices.ca/findhealth/), and snowball sampling. All PN programs identified through the secondary search were contacted for additional information on the types of services offered. PN programs that failed to respond to repeated contact attempts were excluded from the study. Of the 58 PN programs identified in the province‐wide environmental scan, only five met the study's inclusion criteria due to incompatibility with the programs' scope (e.g., did not have a clearly outlined ‘navigator’ position) and/or target population (e.g., did not work with adult patients) [48]. Patient experiences from two of the eligible programs were captured in the study alongside other, more recently established, PN programs. PN program directors and navigators of identified PN interventions acted as key informants, helping to identify and recruit study participants. Adult patients who had participated in an Alberta‐based ‘navigation’ program within the past 2 years for at least 1 month were eligible.

Ethics approval for the study was obtained by the University of Calgary's Conjoint Health Research Ethics Board.

### 2.3 Data Collection

The interview guide (Table S1) was co‐developed with patient partners and grounded in the socio‐ecological model, recognizing that one's interactions with PN programs may be closely linked to their social positioning and various sources of influence [49]. Virtual and in‐person interviews were conducted from November 2023 to February 2024, each lasting up to 1 h, with an average duration of approximately 50 min. For participants with limited English literacy, trained interpreters were utilized to help facilitate interviews. All interviews were conducted by one of two team members (S.R. [female graduate student] and/or K.T. [female clinician researcher]) who were unfamiliar with the study participants, and who both had prior qualitative research experience and training. Audio files for each interview were transcribed verbatim using Rev audio transcription services (rev. com) and reviewed by a study team member (SR) to ensure transcript accuracy [50]. All participant files were given a unique alphanumeric study ID.

### 2.4 Data Analysis

Participant recruitment, data collection and data analysis were undertaken concurrently until sufficient *information power* was achieved [51, 52, 53]. Inductive thematic analysis was selected as the analytic approach for this study and performed using NVivo software [54, 55]. Given ID's non‐prescriptive nature, the study team recognizes that a variety of qualitative research approaches could have been employed to identify meaningful patterns in the data set [38]. Our choice to use thematic analysis was rooted in our objective to go beyond simple descriptions of PN (i.e., purely exploring its semantic meaning) to examine patients' perspectives and understanding of the conceptual meaning of PN [54]. Braun and Clark have published a typology of research questions that are well suited to be examined and explored through a thematic analysis approach; one such type encompasses questions related to ‘the views, perceptions, understandings, perspectives, needs, motivations of particular groups, about particular phenomena, in particular contexts’ [56]. Thematic analysis is, therefore, an approach that we believe aligns well with our research question and objective, as well as our intention to code for both semantic and latent meanings from the data collected. Furthermore, the use of inductive thematic analysis enabled us to generate data‐derived thematic outputs that evolved organically throughout the coding process [56]. Two team members (K.T., S.R. and/or S.B. [female health services researcher]) independently generated initial codes for each transcript. The team met regularly to develop and refine a consolidated coding tree (Figure S1) and set of themes. Following theme generation, additional analyses were performed to ensure the study findings were appropriately interpreted and could directly inform PN program implementation [57, 58]. Through reflexive conversations with patient partners, a unified schema was co‐built that more harmoniously distilled the essence of PN in the province. Participants were not asked to provide feedback on the study findings.

Throughout the research process, researchers utilized reflexive journaling, wrote post‐interview field notes and debriefed with peers to limit the effects of researcher bias and subjectivity [59]. An audit trail was also created, keeping a systematic record of memos to document the evolution and interpretation of themes [60].

## 3 Results

Twenty‐three participants were included in the study (Table 1). The median (IQR) age of participants was 59 (48–67) years, with 12 participants (52%) self‐identifying as women and 17 (74%) identifying as white. Participants worked with a nurse (48%), lay (39%) or social work (13%) navigator for a median (IQR) duration of 7 (3–24) months. Three study participants were non‐English speaking and required the assistance of an interpreter.

**Table 1:** Descriptive statistics of the study participants accessing PN services.

Variable	Categories	Frequency, *n* (%)
Age		
	18–24	3 (13)
	25–64	12 (52)
	65 and up	8 (35)
Gender		
	Women	12 (52)
	Men	11 (48)
Educational attainment		
	Less than high school	5 (22)
	High school diploma	11 (48)
	College and bachelor's degree	6 (26)
	Graduate degree	1 (4)
Employment status		
	Employed	4 (17)
	Unemployed	7 (30)
	On disability leave	6 (26)
	Retired	6 (26)
Navigator type		
	Nurse navigator	11 (48)
	Social work navigator	3 (13)
	Lay navigator	9 (39)
Relationship Duration		
	Less than 3 months	6 (26)
	3 months to 1 year	10 (44)
	More than 1 year	7 (30)

The study participants received services from five distinct PN programs across Alberta. One program was specifically designed for patients receiving chemotherapy, offering referral support and medical care provision (*n* = 8). Two other programs were designed to support transitions of care – one for transitioning from paediatric to adult care (*n* = 3) and the other from hospital to home (*n* = 3). The last two programs were based in the community – one in primary care (*n* = 6) and the other in a community organization that assists individuals who have recently immigrated to Canada (*n* = 3). All programs offered primarily in‐person services, with opportunities for over‐the‐phone correspondence based on participant preference. Participant perceptions of essential PN functions were not found to differ between navigator training backgrounds.

**Figure 1:**
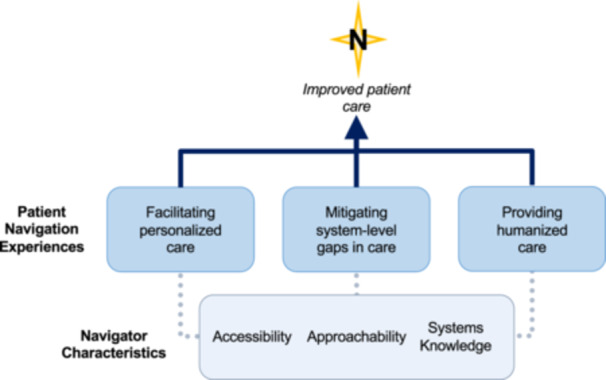
Visual Depiction of Participants' Understanding of PN Functions and Characteristics. Participants' understandings of PN were largely categorized into three function‐based themes surrounding the facilitation of personalized care, the mitigation of system‐level care gaps and the provision of humanized care. These three navigator functions were made possible by three characteristic‐based themes pertaining to navigator accessibility, approachability and systems knowledge. Irrespective of navigator training or navigation setting, the participants' experiences suggest that the programs were further unified in their pursuit of improving perceived patient care.

Six themes were generated that captured participant‐identified functions of PN, as well as the characteristics of PN that were felt to be of particular value and importance in facilitating these functions (Figure 1). Illustrative quotes for function‐ and characteristic‐specific themes are provided in Tables 2 and 3, respectively.

**Table 2:** Illustrative quotes for patient‐perceived navigator functions.

Themes	Quotes
Personalized Care	‘They know me better [after 15 years]. They know my frailties, my bones, my feet. They have never failed to ask me how things are going. That way they understand my outside issues as well as the chemo treatments… They know me better and they are truly interested in their patients.’ (Participant 1 – woman, nurse PN program) ‘[The navigator] definitely helped me find who I was within the healthcare system and my personal life as well. She was able to talk to my doctors if I didn't hear from them… and she kind of kicked them in gear a bit, which was nice, but also knowing that she had my back and she believed me like, ‘Okay, so how can we fix this? How can I help you to become your own independent person? … I want you to find who you are so that I can help you with everything else.’ Yeah, she was just a very positive person and I loved that.’ (Participant 10 – woman, social work PN program) ‘[The navigator] is multitasking what [another clinic] was doing with the different people, right? There was a dietician and counselling and more available, about 3, 4, 5 people there were helping me, but here she's the only one doing lots of stuff.’ (Participant 15 – woman, lay community health PN program) ‘There isn't anything like booking blood tests, booking scans, coming back from surgery, or getting staples removed ‐ anything that I needed could always be taken care of by them.’ (Participant 20 – man, nurse PN program)
Seamless Care	‘Anytime I had appointments, usually with either the different types of doctors or nurses or whatever else, [the navigator] would usually join along. She would listen to it and then afterwards… [would be] able to have that buffer… to put it into words of rather than just like, “Okay, here's the meds,” but “This is why you're taking it. Here's what it's for.”’ (Participant 2 – man, nurse PN program) ‘Any questions that we have or for the oncologist, instead of calling the oncologist's office and having to go through all that, [the navigator would] email them directly and the responses, fortunately, to them are much quicker and then [the navigator] can translate everything. Whereas if we call the office ourselves, there's a little bit more of a lag… The nurse navigators are amazing at helping us to understand what's going on.’ (Participant 6 – man, nurse PN program) ‘She's filling a hole of being discharged from the hospital to your home when you open the door and there's nobody there.’ (Participant 13 – woman, nurse PN program) ‘The doctors, they're a big deal, but they're not there all the time like the navigators are. If I ever had any questions, it was never a problem. I always had their number and could give them a call if you need anything… If they didn't have it, they'd find it for me.’ (Participant 20 – man, nurse PN program)
Humanized Care	‘The person is important to them – that we recognize we are important in this and how all of this impacts us as well… They ask us that if we have any concerns, that it's important to them and that they will help us with anything that we might have a concern about, and they make that clear, and I find that very different from other avenues of medical care.’ (Participant 5 – man, nurse PN program) ‘They're treating you like you are a person, not just a number or just a patient, but you are a person… It's not too often that people get to take the time to get to know you… and so being able to talk to [the one navigator] about Halloween and to talk to [the other] about her [family] for a few minutes, it's nice. You could just have that conversation sort of thing.’ (Participant 8 – woman, nurse PN program) ‘She listened versus lectured.’ (Participant 13 – woman, nurse PN program) ‘It's not like therapy, but it is like a conversation and that's so refreshing.’ (Participant 17 – woman, lay community health PN program) ‘Everybody is treated, and they know everybody, and they know who they are. I've seen people come in and it is like there is a cloud over the top of your head sometimes, and you go in there and they can make the sunshine a little bit.’ (Participant 20 – man, nurse PN program)

**Table 3:** Illustrative quotes for patient‐perceived navigator characteristics.

Themes	Quotes
Accessibility	‘I just want to say they are also really good because they are open outside of the days that they do chemo, and there were several occasions that I just popped into the office because I had questions.’ (Participant 7 – woman, nurse PN program) ‘If I ever had a small question or a concern, I would email her, and it'd be like an instant reply.’ (Participant 12 – woman, social work PN program) ‘When I was at the hotel for two months for any medical appointment, I always had a navigator with me, helping me through the appointment, which made it so much easier for me and it was a very positive experience.’ (Participant 21 – Interpreted – man, lay community health PN program) ‘The [navigators'] phone line is always open and they're like that with everybody. They tell everybody, ‘If you need anything, don't be scared to call’… They have got time for everybody all the time.’ (Participant 20 – man, nurse PN program)
Approachability	‘They're very bubbly and personable and I feel like I can discuss anything with them. If I have a sore throat or even not medical, say, well, you look like you're upset today, what's going on? Or whatever. And you feel like you can talk to them about anything.’ (Participant 1 – woman, nurse PN program) ‘What do I value most? The easiness it was and how she made you feel comfortable asking a question. Somebody else might think it's the stupidest question, so why ask it? You should know the answer. But she never once laughed at my silly question or even my husband's silly question. She answered it truthfully. And I'd like to say with respect to our ignorance on some of these things that we were going through.’ (Participant 4 – woman, nurse PN program) ‘For somebody like myself having… to actually reach out to my [doctor], I mean, I don't know what the actual process is, but I feel like they're busy enough. I don't want to burden them with phone calls, with questions or concerns that I have if I'm having some kind of symptoms or whatever. It was just so much easier to reach out to the nurse navigator, even if she ultimately ended up contacting my oncologist. It was just, for me, it was the comfort of that.’ (Participant 7 – woman, nurse PN program) ‘I thought it would be more the very structured social worker… I was wary of saying anything too personal in the beginning because I just didn't want things written on my record as per what social work would have to. So, being more informal has really, I've loosened up quite a lot and I feel a little more open now and a little more like, “Okay, there is help in the healthcare system.”’ (Participant 17 – woman, lay community health PN program)
Systems Knowledge	‘They're very knowledgeable and very helpful, and they're always there. It seems like they're always there to answer questions.’ (Participant 6 – man, nurse PN program) ‘The biggest thing for me is if I ask a question and someone doesn't have an answer if they go and search for the answer, that makes them higher up in my books, I guess. And she was always like that. If I asked something completely out of the blue, she would be like, “I don't know, but let me get back to you,” and she would always give me an answer no matter what the question was, even if it wasn't related to me [my condition], if it was kind of off topic about me just becoming an adult, she was going to answer the question even if she didn't have an answer, she would figure it out for me.’ (Participant 12 – woman, social work PN program) ‘Sometimes I don't know the system, but I can learn from [the navigator] what are the new things that they can offer. So, whatever they offer me, I can use it.’ (Participant 15 – woman, lay community health PN program) ‘She's very empathetic and has a wealth of knowledge and a lot of, I think, personal experience, which goes a long way… She knows a lot about a lot of things, so if I bring something up that I'm interested in, she's either heard about it or is involved with it in some way.’ (Participant 17 – woman, lay community health PN program)

### 3.1 Themes Relating to the Function(s) of Navigators Within the Healthcare System

#### 3.1.1 Navigators facilitate **personalized care** by identifying, addressing and prioritizing the diverse needs of patients

Participants spoke of their navigators' ability to centre the patient and their distinct care needs in service interactions. By allowing patients greater control over their communication and respecting their care preferences, participants felt the navigators were better able to tailor their support to specific patient needs: ‘They seem to make you the centre… they're making sure that you are being looked after’ (Participant 8 – woman, nurse PN program). The prioritization of patients' particular care needs also meant that navigators would often serve multiple different care roles, offering clinical, emotional, social and physical support, if pertinent, to their patients: ‘There's nothing I've asked that they weren't able to help me with… there's nothing that they haven't been able to accommodate’ (PID 9 – woman, nurse PN program).

The consistent willingness of the navigators to provide individualized care was exemplified by one participant who recalled receiving multiple different modes of support from her navigator, including guidance on obtaining her driver's license, assistance with university applications and coaching on how to strengthen their clinician communication skills ‐ all things that the participant identified as critical to supporting their independence. Alternatively, another participant described how their navigator provided medical guidance (i.e., advice on when to discontinue certain medications) and emotional reassurance, as well as liaised with their oncologist, assisted with the scheduling of blood tests and imaging and helped remove their surgical staples. The adaptability of navigators was contrasted by participant retellings of other healthcare professional interactions, described by some as feling excessively rigid or ‘pigeonholed’ (Participant 2 – male, nurse PN program).

#### 3.1.2 Navigators facilitate **seamless care** by addressing system‐level care gaps in the healthcare system

Participants described the ability of their navigators to help mitigate informational and structural gaps they were experiencing in their care. These gaps pertained specifically to limited provider availability, insufficient appointment lengths and suboptimal patient‐provider and interprofessional communication: ‘There's a lot of stuff that you are kind of just expected to know… [the navigators are] making sure the foundation is there… and filling in any gaps’ (Participant 11 – man, social work PN program).

As a result of these care gaps, patients described feeling overwhelmed by an ‘avalanche of information’ (Participant 6 – man, nurse PN program). Patients highlighted the role of navigators in translating and/or delivering information to make it more digestible for patients: ‘[The navigators] were able to kind of talk you off the ledge and explain to you what's going on…, and who's going to help you, and what you need, and the resources… It's like they're in your corner right from the beginning’ (Participant 20 – man, nurse PN program).

#### 3.1.3 Navigators facilitate **humanized care** through displays of empathy and warmth

Patients frequently highlighted the importance of empathy and compassion in the navigation process. They discussed the strength and depth of the relationships they were able to cultivate with their navigators, describing their navigators as feeling akin to their friends and family: ‘Beyond the expert care that they're trained to do, it's their input in such a friendly, genuine interaction with you. It doesn't sound clinical. It sounds like you're sitting in a living room having your treatment with people who genuinely want to know more about you’ (Participant 5 – man, nurse PN program).

While such humanistic care provision manifested differently for each patient (e.g., offering physical comforts, personal conversations, or words of encouragement), its presence in PN programs consistently allowed the participants to feel regarded as more than their disease or illness. For many, the empathy and compassion of the navigators emboldened participants and steeled their resilience: ‘It's almost like the light in the tunnel, and it gives you that self‐assurance and it kind of keeps you on that path [to recovery]’ (Participant 2 – male, nurse PN program).

### 3.2 Themes Relating to Navigator Characteristics That Facilitated Their Ability to Provide Personalized, Seamless and/or Humanized Care

#### 3.2.1 Patients value accessibility, where navigators are available and flexible to their needs

Participants frequently spoke of their appreciation for how navigators made themselves available to patients in times of need, which were often outside of regular business hours: ‘Because of my condition, sometimes I needed to go to the hospital in the middle of the night and they were able to accommodate me… I value that thing a lot. The flexibility and the understanding and the empathy’ (Participant 22 – Interpreted – man, lay community health PN program). Knowing the participants had a go‐to person with whom they could always contact provided considerable reassurance and security: ‘One of the main things was just having somebody there who I could reach out to… I found that [with the navigator] being around, if I had any questions or thoughts, I could dump them on her and she would get the answer for me… I remember lying in the bed in the hospital thinking… ‘Oh my God,’ this time I didn't really have to worry about [my care] because all of a sudden there was this person who was going to help me’ (Participant 3 – man, nurse PN program).

#### 3.2.2 Patients value approachability, where navigators could connect with patients free of clinically imposed hierarchies

Participants appreciated the ability of navigators to create spaces where they felt at ease and could speak freely. Navigators were felt to be approachable: ‘[The navigator] was bubbly and friendly. Now I think if someone else came in and they were more ‘just the facts, ma'am, just the facts’ … I think it would have been hard to open up… It wasn't like I was anxious about her visits to come or anything… it just made it a lot easier.’ (Participant 16 – woman, lay community health PN program). Some patients juxtaposed the approachability and comfort they felt with their navigators with their hesitation to speak with their usual clinical care providers: ‘You could go to your doctor and ask these questions, but you're going to be heart racing the whole time because obviously, they have so many other patients… [The navigator] was able to do it and [you] know that if you're going to ask this question, no one's going to ridicule you’ (Participant 12 – woman, social work PN program).

#### 3.2.3 Patients value having a navigator with extensive systems knowledge

All participants referred to their navigators as rich sources of system information. Participants explained the value of having someone with extensive hospital‐ and/or community‐based information who could allocate the time needed to address patient questions effectively: ‘As soon as I talked to the nurse navigator, she was giving information, stuff that I didn't have yet, stuff that I needed, stuff that I wanted. That's what I was looking for’ (Participant 7 – woman, nurse PN program). For the participants, having greater access to information increased their ability to utilize the supports around them and, in doing so, bestowed them with greater autonomy to make informed decisions about their health: ‘Having the navigator has been so supremely helpful because it's getting the ideas for alternative services that people, like myself, did not know were out there… I feel better knowing that there is an alternative to the static healthcare system that we're used to’ (Participant 17 – woman, lay community health PN program).

## 4 Discussion

Our study found that, from the patient's perspective, PN consistently offers care that is personalized, seamless and humanized. These PN functions are made possible by the accessibility, approachability and extensive systems knowledge of the navigators themselves. Though the operationalization of these themes varied in response to distinct care settings and participant needs, the essence of navigator functions and characteristics was consistent across interviews.

The expansion and diversification of PN over the past three decades have sparked numerous efforts to identify and synthesize commonalities across contemporary PN programs. In one such project, Champ and Dixon [61] provide an extensive summary of oncology PN programs across Canada, noting that, while similarities exist regarding programs' methods of service delivery (e.g., the incorporation of virtual care in most models) and targeted age ranges (e.g., primarily adult patients), considerable variation was found in their patient demographics, navigator training, scope of practice and patient referral processes. Similarly, a mixed‐methods study examining Cancer Care Alberta's PN programs showed that, despite the evolution and prevalence of PN programs, gaps relating to navigator role ambiguity continue to cause inconsistencies in program delivery [62]. These studies, together with a recent proceedings paper by the Canadian Healthcare Navigation Conference, underscore the continued lack of clarity surrounding PN, including how PN services differ from the roles and supports provided by other members of the healthcare team [63]. The lack of role and conceptual clarity surrounding PN continues to pose significant barriers to the uptake, implementation and appreciation of PN programs today [64, 65, 66]. In response, Canadian stakeholders have called for greater PN standardization, recognizing that the current lack of a shared vision for navigation programs limits the positive impact that PN programs can have [63].

To address this lack of clarity, multiple reviews of PN have been conducted in different PN care fields (i.e., oncology, dementia and mental health and addiction) to better understand not only the operations and characteristics of PN but also their *functions* and *core themes* [66, 67, 68]. Although these reviews identified similar PN functions, specifically as they relate to care coordination and relationship building, their interpretations of these thematic concepts varied. For instance, the notion of ‘care coordination’ was described by Katerenchuk and Salas [68] to mean supporting all patient health needs, disseminating medical and nonmedical information, and remaining available throughout their care journey. In contrast, Anthonisen et al. [66] described ‘care coordination’ more literally as navigators facilitating patient access to pertinent services and resources. Relationship‐building was also interpreted differently across these reviews, especially with regard to the depth, extent and purpose of the relationships formed. For example, Mullen et al. [67] promoted the use of navigational teams (i.e., navigators working collaboratively to support patient needs) while others advocated for one‐on‐one patient‐navigator relationships, describing the importance of relationship consistency in counteracting patients' other, often fragmented, connections to clinical support [68]. While these reviews offer a more comprehensive understanding of PN than program‐specific studies, the nuanced differences in their findings and their positioning in specific clinical contexts make it difficult to deduce what PN functions are cross‐contextual and therefore potentially fundamental to the PN construct itself. Although this qualitative study does not, and cannot, aim to definitively identify these fundamental components of the broader PN construct, it does elucidate elements of the intervention that patients feel are of critical importance.

Our study builds upon the findings of these PN studies by integrating patient perspectives across operationally and contextually distinct PN programs. While care coordination was repeatedly identified as an important PN function in prior reviews, our findings suggest that navigator functions extend beyond traditional ‘coordination’ (i.e., connecting patients across care providers, systems and services) to the creation of seamless care experiences more broadly for patients. ‘Seamless care’ is characterized by navigators working to fill gaps in patient care (e.g., patients referred to the ability of their navigators to address gaps related to the use of medical jargon, inefficient access to the broader medical team and abrupt hospital discharges), which resulted in reduced patient stress and increased feelings of comfort in their care. Therefore, while seamless care certainly incorporates aspects of traditional care coordination, this theme encompasses a broader component of PN that better resonates with patients' understanding of PN. As was also seen in prior reviews, our findings highlight the importance of relationship‐building between the navigator and the patient. With that said, we note that study participants from different PN programs often described different manifestations of the patient‐navigator relationship. For example, some described a deep connection with the navigator and valued the emotional support provided, while others valued the relational aspects of PN, even without this depth of relationship. Regardless of how the patient‐navigator relationship was operationalized across programs, its perceived value and importance was rooted in its ability to make the patient feel regarded as more than their illness. We therefore broaden the idea of relationship building to better address the provision of ‘humanized care’ as a PN function.

Taken together, the three PN functions identified in our study appear to collectively compensate for the existing systemic and structural holes in contemporary healthcare systems. The current stresses and fraying of our healthcare systems have made the time, resources and flexibility needed to provide personalized, seamless and humanized care by healthcare teams increasingly untenable [69]. In this study, participants consistently noted the importance of navigators in providing personalized care, in part, because of the difficulty they faced receiving such customization from other care providers. Similarly, the ubiquity with which participants spoke of their navigators' ability to create seamless care experiences was, at times, a direct response to their experiences with increasingly fragmented care pathways [70]. Overall, the commonalities in participant understandings of distinct PN programs suggest key points of overlap in PN that may be indicative of broader PN tenets. This study also highlights the value patients ascribe to PN as a means of patching system‐level problems that have led to such rigid and fragmented care experiences.

### 4.1 Strengths and Limitations

The study's use of maximum variation purposive sampling enabled the research team to collate experiences from patients of diverse backgrounds and distinct PN program interactions. The heterogeneity present across our participant sample strengthened the credibility of our findings, as well as permitted us to further examine the nuances of patient interactions with PN programs. Additionally, the involvement of our patient partners was crucial in anchoring our work and ensuring our findings resonated with both researchers and patients alike.

Limitations to this project include the possibility that, given our use of key informants in participant recruitment, those with negative PN experiences may have been dissuaded from participating or may have never been contacted. This has the potential to bias our findings to be overly positive, blurring elements of PN that may need modification for broader patient utilization or greater receptivity to the service. While such a bias may be present, it is worth reiterating that the purpose of this study was not to explore patients' satisfaction with PN services, nor to evaluate their effectiveness, but to identify the key functions and characteristics of these programs, should they exist. Additionally, while the participant sample generally displayed a high degree of heterogeneity, it was relatively homogenous in terms of participants' race and ethnicity, with nearly 75% of the sample self‐identifying as white. Moving forward, greater participant diversity should be sought with respect to participant race or ethnicity to provide greater insight into the culturally tailored possibilities of PN. Lastly, several types of PN programs were not included in this project. While this study provides insight on how a subset of patients conceptualize PN programs, future research that explores patient experiences with programs specializing in different care domains (e.g., dementia, mental health, HIV, or autism), with navigators of different training backgrounds (e.g., pharmacists or peer navigators), or with Indigenous‐specific PN programs may elucidate additional navigator functions and/or characteristics not directly captured in this study. Furthermore, because PN programs for this study were primarily identified via publicly available program websites and an Alberta Health Services directory, we likely missed programs that were either (i) not funded, administered, or delivered by Alberta Health Services, or (ii) did not have websites for the public (e.g., smaller programs or those that did not permit self‐referrals). The limited number of PN programs that were ultimately represented may indicate that there are additional PN functions and characteristics beyond those captured in this study, or that further nuances may need to be considered for the functions that were identified. Although an exhaustive overview of patients' understanding of PN was never the intent of this study, this does likely restrict the transferability of our findings.

## 5 Conclusion

This study extends current knowledge about the commonalities that exist across distinct PN programs, from the patient's perspective. The findings indicate

that patients repeatedly understand PN services as those which offer personalized, seamless and humanized care; and that they deeply value navigators' accessibility, approachability and extensive systems knowledge. This study suggests that, despite increasing heterogeneity in how PN programs are designed and operationalized, key irreducible functions and characteristics of PN continue to persist across contemporary programs.

## Author Contributions

**Sarah Rabi:** conceptualization, project administration, methodology, investigation, validation, data curation, formal analysis, visualization, writing – Original draft. **Maria Santana:** supervision, conceptualization, methodology, investigation, writing – reviewing and editing. **Gina Dimitropoulos:** supervision, conceptualization, methodology, writing – reviewing and editing. **Kerry McBrien:** supervision, conceptualization, methodology, writing – reviewing and editing. **Eleanor Benterud:** methodology, investigation, formal analysis, visualization, writing – reviewing and editing. **Lorraine Wigston:** methodology, investigation, formal analysis, visualization, writing – reviewing and editing. **Karen Tang:** funding acquisition, supervision, conceptualization, project administration, methodology, investigation, validation, formal analysis, visualization, writing – reviewing and editing.

## Ethics Statement

Ethics approval for the study was obtained by the University of Calgary's Conjoint Health Research Ethics Board.

## Conflicts of Interest

The authors declare no conflicts of interest.

## Supporting information

PN_Patient_Experiences_Supplement_Revised.

## Data Availability

While we will be sharing our interview guide and coding scheme, we will not be making participant transcripts available in an effort to protect participant privacy and confidentiality.

## References

[1] H. P. Freeman , B. J. Muth , and J. F. Kerner , “Expanding Access to Cancer Screening and Clinical Follow‐Up Among the Medically Underserved,” Cancer Practice 3, no. 1 (1995): 19–30. 7704057

[2] H. P. Freeman , “A Model Patient Navigation Program,” Oncology Issues 19, no. 5 (2004): 44–46, 10.1080/10463356.2004.11884227.

[3] H. P. Freeman and R. L. Rodriguez , “History and Principles of Patient Navigation,” Cancer 117, no. 15 0 (2011): 3539–3542, 10.1002/cncr.26262. 21780088 PMC4557777

[4] H. P. Freeman , “Patient Navigation: A Community Based Strategy to Reduce Cancer Disparities,” Journal of Urban Health 83, no. 2 (2006): 139–141, 10.1007/s11524-006-9030-0. 16736361 PMC2527166

[5] S. F. Oluwole , A. O. Ali , A. Adu , et al., “Impact of a Cancer Screening Program on Breast Cancer Stage at Diagnosis in a Medically Underserved Urban Community,” Journal of the American College of Surgeons 196, no. 2 (2003): 180–188, 10.1016/S1072-7515(02)01765-9. 12595043

[6] V. A. Parker , J. A. Clark , J. Leyson , et al., “Patient Navigation: Development of a Protocol for Describing What Navigators Do,” Health Services Research 45, no. 2 (2010): 514–531, 10.1111/j.1475-6773.2009.01079.x. 20132342 PMC2838158

[7] K. A. McBrien , N. Ivers , L. Barnieh , et al., “Patient Navigators for People With Chronic Disease: A Systematic Review,” PLoS One 13, no. 2 (2018): e0191980, 10.1371/journal.pone.0191980. 29462179 PMC5819768

[8] I. V. Bassett , S. M. Coleman , J. Giddy , et al., “Sizanani: A Randomized Trial of Health System Navigators to Improve Linkage to HIV and TB Care in South Africa,” JAIDS Journal of Acquired Immune Deficiency Syndromes 73, no. 2 (2016): 154–160, 10.1097/QAI.0000000000001025. 27632145 PMC5026386

[9] T. M. English , D. Masom , and M. V. Whitman , “The Impact of Patient Navigation on Diabetes,” Journal of Healthcare Management/American College of Healthcare Executives 63, no. 3 (2018): 32, 10.1097/JHM-D-16-00033. 29734289

[10] J. E. Anderson and S. C. Larke , “The Sooke Navigator Project: Using Community Resources and Research to Improve Local Service for Mental Health and Addictions,” Mental Health in Family Medicine 6, no. 1 (2009): 21–28. 22477884 PMC2777592

[11] L. G. Jiang , Y. Zhang , E. Greca , et al., “Emergency Department Patient Navigator Program Demonstrates Reduction in Emergency Department Return Visits and Increase in Follow‐Up Appointment Adherence,” American Journal of Emergency Medicine 53 (2022): 173–179, 10.1016/j.ajem.2022.01.009. 35065524

[12] E. Samuels , L. Kelley , T. Pham , et al., “‘I Wanted to Participate in My Own Care’: Evaluation of a Patient Navigation Program,” Western Journal of Emergency Medicine 22, no. 2 (2021): 417–426, 10.5811/westjem.2020.9.48105. 33856334 PMC7972383

[13] N. Carter , R. K. Valaitis , A. Lam , J. Feather , J. Nicholl , and L. Cleghorn , “Navigation Delivery Models and Roles of Navigators in Primary Care: A Scoping Literature Review,” BMC Health Services Research 18, no. 1 (2018): 96, 10.1186/s12913-018-2889-0. 29422057 PMC5806255

[14] J. Bhawra , A. Toulany , E. Cohen , C. Moore Hepburn , and A. Guttmann , “Primary Care Interventions to Improve Transition of Youth With Chronic Health Conditions From Paediatric to Adult Healthcare: A Systematic Review,” BMJ Open 6, no. 5 (2016): e011871, 10.1136/bmjopen-2016-011871. PMC4861092 27150188

[15] P. Y. Chu , G. R. Maslow , M. von Isenburg , and R. J. Chung , “Systematic Review of the Impact of Transition Interventions for Adolescents With Chronic Illness on Transfer From Pediatric to Adult Healthcare,” Journal of Pediatric Nursing 30, no. 5 (2015): 19–27, 10.1016/j.pedn.2015.05.022. PMC4567416 26209872

[16] D. San Martin‐Feeney , S. Samborn , B. Allemang , et al., “Transition Experiences of Adolescents and Young Adults Working With a Patient Navigator,” Health Care Transitions 3 (2025): 100088, 10.1016/j.hctj.2024.100088. 39712475 PMC11658225

[17] A. E. Reid , S. Doucet , and A. Luke , “Exploring the Role of Lay and Professional Patient Navigators in Canada,” Journal of Health Services Research & Policy 25, no. 4 (2020): 229–237, 10.1177/1355819620911679. 32188293

[18] L. Fillion , S. Cook , A. M. Veillette , et al., “Professional Navigation Framework: Elaboration and Validation in a Canadian Context,” Oncology Nursing Forum 39 (2012): E58–E69, 10.1188/12.ONF.E58-E69. 22201669

[19] T. Greenhalgh , G. Robert , F. Macfarlane , P. Bate , and O. Kyriakidou , “Diffusion of Innovations in Service Organizations: Systematic Review and Recommendations,” Milbank Quarterly 82, no. 4 (2004): 581–629, 10.1111/j.0887-378X.2004.00325.x. 15595944 PMC2690184

[20] J. L. Denis , Y. Hébert , A. Langley , D. Lozeau , and L. H. Trottier , “Explaining Diffusion Patterns for Complex Health Care Innovations,” Health Care Management Review 27, no. 3 (2002): 60–73, 10.1097/00004010-200207000-00007. 12146784

[21] V. A. Parker and C. H. Lemak , “Navigating Patient Navigation: Crossing Health Services Research and Clinical Boundaries,” Advanced Health Care Management 11 (2011): 149–183, 10.1108/s1474-8231(2011)0000011010. 22908669

[22] M. K. Anderson , M. J. Reff , R. S. McMahon , and D. R. Walters , “The Role of the Oral Oncology Nurse Navigator: Oral Oncology Nurse Navigators Improve Patient Care & Satisfaction,” Oncol Issues 32, no. 5 (2017): 26–30, 10.1080/10463356.2017.11905287.

[23] R. E. Gearing , N. El‐Bassel , A. Ghesquiere , S. Baldwin , J. Gillies , and E. Ngeow , “Major Ingredients of Fidelity: A Review and Scientific Guide to Improving Quality of Intervention Research Implementation,” Clinical Psychology Review 31, no. 1 (2011): 79–88, 10.1016/j.cpr.2010.09.007. 21130938

[24] T. J. Horton , J. H. Illingworth , and W. H. P. Warburton , “Overcoming Challenges in Codifying and Replicating Complex Health Care Interventions,” Health Affairs 37, no. 2 (2018): 191–197, 10.1377/hlthaff.2017.1161. 29401020

[25] K. B. Roland , D. H. Higa , C. A. Leighton , Y. Mizuno , J. B. DeLuca , and L. J. Koenig , “Client Perspectives and Experiences With HIV Patient Navigation in the United States: A Qualitative Meta‐Synthesis,” Health Promotion Practice 21, no. 1 (2020): 25–36, 10.1177/1524839919875727. 31597497 PMC6917848

[26] S. F. Gallups , J. Demirci , M. Nilsen , J. Burke , C. Bender , and M. Q. Rosenzweig , “Using Concept Mapping to Explore Interpersonal Communication Components of Patient Navigation in Breast Cancer Care,” Cancer Nursing 46, no. 4 (2023): 321–330, 10.1097/NCC.0000000000001118. 35439221 PMC11182702

[27] J. K. Carroll , S. G. Humiston , S. C. Meldrum , et al., “Patients' Experiences With Navigation for Cancer Care,” Patient Education and Counseling 80, no. 2 (2010): 241–247, 10.1016/j.pec.2009.10.024. 20006459 PMC2891343

[28] L. Gotlib Conn , M. Hammond Mobilio , O. D. Rotstein , and S. Blacker , “Cancer Patient Experience With Navigation Service in an Urban Hospital Setting: A Qualitative Study,” European Journal of Cancer Care 25, no. 1 (2016): 132–140, 10.1111/ecc.12247. 25295379

[29] A. Wilkinson , J. Atlas , K. Nelson , and H. Mulligan , “Client Perceptions of Engaging With a Health and Social Care Navigation Service: A Qualitative Study,” Chronic illness 18, no. 1 (2022): 169–180, 10.1177/1742395320937046. 32727202

[30] C. H. H. Tan , S. Wilson , and R. McConigley , “Experiences of Cancer Patients in a Patient Navigation Program: A Qualitative Systematic Review,” JBI Database of Systematic Reviews and Implementation Reports 13, no. 2 (2015): 136–168, 10.11124/jbisrir-2015-1588. 26447039

[31] A. P. Hudson , A. J. Spooner , N. Booth , et al., “Qualitative Insights of Patients and Carers Under the Care of Nurse Navigators,” Collegian 26, no. 1 (2019): 110–117, 10.1016/j.colegn.2018.05.002.

[32] A. Luke , K. E. Luck , and S. Doucet , “Experiences of Caregivers as Clients of a Patient Navigation Program for Children and Youth With Complex Care Needs: A Qualitative Descriptive Study,” International Journal of Integrated Care 20, no. 4 (2020): 10, 10.5334/ijic.5451. PMC7664297 33250675

[33] H. Palomino , D. Peacher , E. Ko , S. I. Woodruff , and M. Watson , “Barriers and Challenges of Cancer Patients and Their Experience With Patient Navigators in the Rural US/Mexico Border Region,” Journal of Cancer Education 32, no. 1 (2017): 112–118, 10.1007/s13187-015-0906-0. 26362872

[34] K. Hede , “Agencies Look to Patient Navigators To Reduce Cancer Care Disparities,” JNCI: Journal of the National Cancer Institute 98, no. 3 (2006): 157–159, 10.1093/jnci/djj059. 16449671

[35] P. Hawe , “Lessons From Complex Interventions to Improve Health,” Annual Review of Public Health 36, no. Volume 36, 2015 (2015): 307–323, 10.1146/annurev-publhealth-031912-114421. 25581153

[36] P. Hawe , A. Shiell , and T. Riley , “Complex Interventions: How Out of Control’ Can a Randomised Controlled Trial Be?,” BMJ 328, no. 7455 (2004): 1561–1563. 15217878 10.1136/bmj.328.7455.1561 PMC437159

[37] J. Thompson Burdine , S. Thorne , and G. Sandhu , “Interpretive Description: A Flexible Qualitative Methodology for Medical Education Research,” Medical Education 55, no. 3 (2021): 336–343, 10.1111/medu.14380. 32967042

[38] S. E. Thorne , Interpretive Description: Qualitative Research for Applied Practice (Routledge, 2016). 2nd edition, 10.4324/9781315545196.

[39] S. Thorne , S. R. Kirkham , and J. MacDonald‐Emes , “Interpretive Description: A Noncategorical Qualitative Alternative for Developing Nursing Knowledge,” Research in Nursing & Health 20, no. 2 (1997): 169–177, 10.1002/(sici)1098-240x(199704)20:2<169::aid-nur9>3.0.co;2-i. 9100747

[40] S. Thorne , S. R. Kirkham , and K. O'flynn‐Magee , “The Analytic Challenge in Interpretive Description,” International Journal of Qualitative Methods 3, no. 1 (2004): 1–11, 10.1177/160940690400300101.

[41] J. Ponterotto , “Brief Note on the Origins, Evolution, and Meaning of the Qualitative Research Concept Thick Description,” Qualitative Report 11, no. 3 (2006): 538–549, 10.46743/2160-3715/2006.1666.

[42] M. Warren , T. Leamon , A. Hall , et al., “The Role of Patient Advisory Councils in Health Research: Lessons From Two Provincial Councils in Canada,” Journal of Patient Experience 7, no. 6 (2020): 898–905, 10.1177/2374373520909598. 33457517 PMC7786741

[43] P. M. Pittman , “Gendered Experiences of Health Care,” International Journal for Quality in Health Care 11, no. 5 (1999): 397–405, 10.1093/intqhc/11.5.397. 10561031

[44] A. E. Thompson , Y. Anisimowicz , B. Miedema , W. Hogg , W. P. Wodchis , and K. Aubrey‐Bassler , “The Influence of Gender and Other Patient Characteristics on Health Care‐Seeking Behaviour: A QUALICOPC Study,” BMC Family Practice 17, no. 1 (2016): 38, 10.1186/s12875-016-0440-0. 27036116 PMC4815064

[45] J. Adamson , Y. Ben‐Shlomo , N. Chaturvedi , and J. Donovan , “Ethnicity, Socio‐Economic Position and Gender‐‐Do They Affect Reported Health‐Care Seeking Behaviour?,” Social Science & Medicine (1982) 57, no. 5 (2003): 895–904, 10.1016/s0277-9536(02)00458-6. 12850114

[46] A. Rankin , A. Baumann , B. Downey , R. Valaitis , A. Montour , and P. Mandy , “The Role of the Indigenous Patient Navigator: A Scoping Review,” Canadian Journal of Nursing Research 54, no. 2 (2022): 199–210, 10.1177/08445621211066765. PMC9109580 35014886

[47] Government of Canada , TCPS 2 (2022) – Chapter 9: Research Involving the First Nations, Inuit, and Métis Peoples of Canada, Panel on Research Ethics, January 11, 2023, accessed June 19, 2025, https://ethics.gc.ca/eng/tcps2-eptc2_2022_chapter9-chapitre9.html.

[48] K. L. Tang , J. Kelly , N. Sharma , and W. A. Ghali , “Patient Navigation Programs in Alberta, Canada: An Environmental Scan,” CMAJ Open 9, no. 3 (2021): E841–E847, 10.9778/cmajo.20210004. PMC8428899 34493550

[49] J. F. Sallis , N. Owen , and E. Fisher , “Ecological Models of Health Behavior.” Health Behavior and Health Education (John Wiley & Sons, 2008), 4th ed., 465–485.

[50] Fast, Accurate Transcription Service , accessed June 9, 2023, https://www.rev.com/.

[51] K. Vasileiou , J. Barnett , S. Thorpe , and T. Young , “Characterising and Justifying Sample Size Sufficiency in Interview‐Based Studies: Systematic Analysis of Qualitative Health Research Over a 15‐Year Period,” BMC Medical Research Methodology 18, no. 1 (2018): 148, 10.1186/s12874-018-0594-7. 30463515 PMC6249736

[52] V. Braun and V. Clarke , “To Saturate or Not to Saturate? Questioning Data Saturation as a Useful Concept for Thematic Analysis and Sample‐Size Rationales,” Qualitative Research in Sport, Exercise and Health 13, no. 2 (2021): 201–216, 10.1080/2159676X.2019.1704846.

[53] J. Low , “A Pragmatic Definition of the Concept of Theoretical Saturation,” Sociological Focus 52, no. 2 (2019): 131–139, 10.1080/00380237.2018.1544514.

[54] V. Braun and V. Clarke , “Using Thematic Analysis in Psychology,” Qualitative Research in Psychology 3, no. 2 (2006): 77–101, 10.1191/1478088706qp063oa.

[55] V. Braun and V. Clarke , “What Can ‘Thematic Analysis’ Offer Health and Wellbeing Researchers?,” International Journal of Qualitative Studies on Health and Well‐Being 9, no. 1 (2014): 26152, 10.3402/qhw.v9.26152. 25326092 PMC4201665

[56] V. Braun and V. Clarke , “Conceptual and Design Thinking for Thematic Analysis,” Qualitative Psychology 9, no. 1 (2022): 3–26, 10.1037/qup0000196.

[57] S. Thorne , Data Analysis Using Interpretive Description Methodology. Presented at: IQRT Webinar Series; July 27, 2022, accessed June 25, 2023, https://www.youtube.com/watch?v=2Xbomla9W8U.

[58] M. A. Neergaard , F. Olesen , R. S. Andersen , and J. Sondergaard , “Qualitative Description – the Poor Cousin of Health Research?,” BMC Medical Research Methodology 9, no. 1 (2009): 52, 10.1186/1471-2288-9-52. 19607668 PMC2717117

[59] J. Green and N. Thorogood , Qualitative Methods for Health Research (SAGE Publications Ltd, 2018). 4th ed..

[60] B. C. O'Brien , I. B. Harris , T. J. Beckman , D. A. Reed , and D. A. Cook , “Standards for Reporting Qualitative Research: A Synthesis of Recommendations,” Academic Medicine 89, no. 9 (2014): 1245–1251, 10.1097/ACM.0000000000000388. 24979285

[61] S. Champ and C. Dixon , “Cancer Patient Navigation in Canada: Directions From the North,” Seminars in Oncology Nursing 40, no. 2 (2024): 151588, 10.1016/j.soncn.2024.151588. 38331627

[62] L. Watson , S. M. Anstruther , C. Link , et al., “Enhancing Cancer Patient Navigation: Lessons From an Evaluation of Navigation Services in Alberta, Canada,” Current Oncology 32, no. 5 (2025): 287, 10.3390/curroncol32050287. 40422546 PMC12110542

[63] R. Markoulakis , A. Luke , A. Reid , K. Mehra , A. Levitt , and S. Doucet , “Proceedings of the Inaugural Canadian Healthcare Navigation Conference: A Forum for Sharing Innovations and Best Practices in Navigation Services,” BMC Proceedings 15, no. 16 (2021): 24, 10.1186/s12919-021-00229-0. 34844595 PMC8629593

[64] K. M. Kokorelias , S. Gould , T. Das Gupta , N. Ziegler , D. Cass , and S. L. Hitzig , “Implementing Patient Navigator Programmes Within a Hospital Setting in Toronto, Canada: A Qualitative Interview Study,” Journal of Health Services Research & Policy 27, no. 4 (2022): 313–320, 10.1177/13558196221103662. 35593462

[65] S. Doucet , A. Luke , and G. Anthonisen , “Hospital‐Based Patient Navigation Programs for Patients Who Experience Injury‐Related Trauma and Their Caregivers: A Scoping Review,” BMJ Open 12, no. 12 (2022): e066260, 10.1136/bmjopen-2022-066260. PMC9806040 36572494

[66] G. Anthonisen , A. Luke , L. MacNeill , A. L. MacNeill , A. Goudreau , and S. Doucet , “Patient Navigation Programs for People With Dementia, Their Caregivers, and Members of the Care Team: A Scoping Review,” JBI Evidence Synthesis 21, no. 2 (2022): 281–325, 10.11124/JBIES-22-00024. PMC10578521 36449660

[67] J. N. Mullen , A. Levitt , and R. Markoulakis , “Supporting Individuals With Mental Health and/Or Addictions Issues Through Patient Navigation: A Scoping Review,” Community Mental Health Journal 59, no. 1 (2023): 35–56, 10.1007/s10597-022-00982-2. 35648257

[68] J. Katerenchuk and A. Santos Salas , “An Integrative Review on the Oncology Nurse Navigator Role in the Canadian Context,” Canadian Oncology Nursing Journal 33, no. 4 (2023): 385–399, 10.5737/23688076334385. 38919590 PMC11195828

[69] S. A. Nancarrow , “Six Principles to Enhance Health Workforce Flexibility,” Human Resources for Health 13, no. 1 (2015): 9, 10.1186/1478-4491-13-9. 26264184 PMC4532254

[70] K. C. Stange , “The Problem of Fragmentation and the Need for Integrative Solutions,” Annals of Family Medicine 7, no. 2 (2009): 100–103, 10.1370/afm.971. 19273863 PMC2653966

