# Supplementary material for: Exploring Patient Understandings of Navigation Services Within Alberta's Healthcare System: A Qualitative Study
Source: Health Expect. 2025 Aug 11;28(4):e70383. doi: 10.1111/hex.70383 (PMC12339916; doi:10.1111/hex.70383)
Supplement: Supplementary file 1 — PN_Patient_Experiences_Supplement_Revised. [file HEX-28-e70383-s001.docx]

**Supplementary Material**

[eTable 1. Interview Question Guide 2](#_Toc176688199)

[eFigure B. Coding Tree Generated Prior to Thematic Analysis 4](#_Toc176688200)

# **eTable 1. Interview Question Guide**

| **Interview Segments** | **Interview Script/Questions** |
| --- | --- |
| **Preamble** | Thank you very much for agreeing to take part in this research study and sharing some of your personal experiences surrounding patient navigation. Throughout the interview, I will ask you questions about your interactions with your patient navigator as well as your reflections on the services provided to you.  The purpose of this interview is to better grasp your experiences with, and understanding of, patient navigation, as well as identify the features of patient navigation services that are most important to you.  If you have any questions or concerns throughout the interview, please let me know. |
| **Questions about previous experiences with PN** | 1. Can you describe your patient navigation experience?    - How did you come to hear about the program?    - What did the navigator do for you?    - When and how did you interact with your navigator?      - Was it through email, in-person meetings, phone calls, etc.?      - What was the frequency of the meetings?      - Did you have regular meetings with them or just reach out when you needed them?    - How long were you in the patient navigation program for? 2. What was positive and/or negative about the experience? |
| **Questions about the impact of PN** | - 1. Have the patient navigators made a difference to your health? - Has it improved your self-efficacy or ability to take care of yourself?   1. Has it made a difference to how you engage with the healthcare system or other care providers? - Do you find it easier or harder to speak to other clinicians? - Has it changed your level of trust with the healthcare system?   1. What did you value most about the navigator?   2. Were there any services you were hoping to receive from the navigator which could not be done? |
| **Questions about the optimized PN role** | 1. How much did you know about patient navigation before your experience with it? 2. What expectations did you have when you started working with the navigator?    - Did they meet your expectations?   IF the participant did not know what patient navigation was…   - How did the navigators introduce themselves to you? - When you were referred to the navigators, did you have any expectations of their role?  1. If someone who has never heard of patient navigation were to ask you about it, how would you explain it to them? |

# **eFigure B. Coding Tree Generated Prior to Thematic Analysis**
